# Risk factors and clinical and laboratory findings associated with feline immunodeficiency virus and feline leukemia virus infections in Bangkok, Thailand

**DOI:** 10.14202/vetworld.2022.1601-1609

**Published:** 2022-07-05

**Authors:** Oumaporn Rungsuriyawiboon, Thitichai Jarudecha, Supa Hannongbua, Kiattawee Choowongkomon, Chaiwat Boonkaewwan, Jatuporn Rattanasrisomporn

**Affiliations:** 1 Department of Veterinary Technology, Faculty of Veterinary Technology, Kasetsart University, Chatuchak, Bangkok 10900, Thailand; 2 Department of Chemistry, Faculty of Science, Kasetsart University, Chatuchak, Bangkok 10900, Thailand; 3 Department of Biochemistry, Faculty of Science, Kasetsart University, Chatuchak, Bangkok 10900, Thailand; 4 Akkhraratchakumari Veterinary College, Walailak University, Nakhon Si Thammarat 80161, Thailand; 5 Department of Companion Animal Clinical Sciences, Faculty of Veterinary Medicine, Kasetsart University, Chatuchak, Bangkok 10900, Thailand

**Keywords:** feline immunodeficiency virus, feline leukemia virus, hematology, risk factors, serum biochemistry

## Abstract

**Background and Aim:**

Feline leukemia virus (FeLV) and feline immunodeficiency virus (FIV) are retroviruses associated with chronic and neoplastic diseases in domestic and non-domestic cats. There has been increasing interest in the clinical importance of feline retroviruses in Thailand and the identification of associated risk factors in domestic cats. To prevent the spread of retroviral diseases and improve the management of retrovirus-infected cats, risk factors and associated clinical laboratory data must be clearly understood. This study aimed to identify the influence of household, lifestyle, health status, sterilization, clinical presentations, and laboratory findings on FIV- and FeLV-infected cats in Bangkok, Thailand.

**Materials and Methods:**

A total of 480 cats were evaluated for FeLV p27 antigen and FIV antibodies using Witness FeLV-FIV Rapid Test and SNAP FIV/FeLV Combo Test at a veterinary hospital service.

**Results:**

Of the 480 cats tested, 113 were positive for virus infection, including 60 for FeLV (12.5%), 40 for FIV (8.3%), and 13 for both FeLV and FIV (2.7%). The findings revealed that the risk factors for cats infected with FeLV, FIV, or both FeLV and FIV were significantly different compared with those for non-infected cats (p < 0.05). Multivariate analysis showed that multi-cat ownership is a risk factor for the high prevalence of feline retrovirus infection, as multi-cat households exhibited a higher prevalence of infection than single-cat households. Anemic and sick cats were also at a greater risk of testing positive for specific retrovirus infections. FeLV-infected cats had a higher risk of anemia and low erythrocyte and thrombocyte counts (p ≤ 0.0001), whereas FIV-infected cats were more likely to have anemia and leukocytopenia than controls.

**Conclusion:**

Knowledge of the risk factors for retroviral diseases and associated clinical and laboratory findings can be used to develop strategies to reduce FIV and FeLV infections in cats.

## Introduction

Feline immunodeficiency virus (FIV) and feline leukemia virus (FeLV) are retroviruses found in domestic cats worldwide [1]. Horizontal transmission through saliva or other body fluids and vertical transmission have been observed in both viruses [2, 3]. FeLV is a g-retrovirus, whereas FIV is classified as a lentivirus. Although FeLV and FIV are closely associated with infections, their potential to cause harm may vary [4]. For FeLV testing, most cats are screened using in-practice test kits based on the detection of p27, one of the major core proteins. Enzyme-linked immunosorbent assays (ELISAs) are widely used for the serodiagnosis of FIV and FeLV; however, results should be confirmed using polymerase chain reaction (PCR) due to false or non-interpretable results [5–7].

FeLV and FIV infections have been reported worldwide and are associated with various symptoms; however, the prevalence of both infections is highly variable among countries and regions [8, 9]. The prevalence of FIV is 2.5–5.2% in the United States and 4.3% in Canada [8, 9]. In Europe, the prevalence of FIV has been extremely high at 13.1% in Hungary, 9.5% in Turkey, and 6.6% in Italy [5, 10, 11]. In Australia and New Zealand, the prevalence has been reported as 2% and 13.7%, respectively [10, 12]. In Asia, the prevalence was 31.3% in Malaysia, 22% in Vietnam, and 5.8% in Thailand [13–15]. The prevalence of FeLV ranges from 2.3% to 3.4% in North America, 15% in Australia, and <1–15% in Europe [8, 9, 16]. In Asia, the prevalence of FeLV was reported as <1–24.5%, and recently in Thailand, the prevalence was reported as 4.2–16.5% [13, 17]. The prevalence of FeLV-FIV coinfection was reported as 0.3% in North America, 4.3% in Malaysia, and 3.5% in Thailand [9, 14, 17]. Several studies have shown a higher overall prevalence of FIV and FeLV in Asia than in Europe or the United States [13].

Infections with FIV and FeLV have similar characteristics, such as being dangerous, challenging, and incurable diseases, but the clinical presentations differ depending on the disease stage. FIV can cause an acquired immunodeficiency syndrome that increases the risk of opportunistic infections, neurological disorders, and tumors [18]. FeLV commonly causes anemia or lymphoma, whereas FIV attacks the immune system and is the leading cause of death in cases with no treatment [16, 19]. There are increasing reports of FIV and FeLV infections causing serious illness and leading todeath in infected cats [2, 20].

Determining the hematologic and clinical chemistry parameters that are characteristic of disease progression would allow a better representation of the asymptomatic stage and a better understanding of the pathogenesis of the two infections [20]. Several studies have reported risk factors for retroviral diseases, including sex, age, aggressive behavior, and outdoor access [12, 13, 21].

Therefore, the present study aimed to characterize the clinical, hematologic, and biochemical parameters in cats diagnosed with FIV and FeLV and identify possible risk factors associated with the infection of the Thai cat population.

## Materials and Methods

### Ethical approval and Informed consent

The study was approved by the Ethics Committee of Kasetsart University (ID# ACKU 63-VTN-007), and cats were included in the study only after written consent was received from their owners.

### Study period and location

The study was conducted from January to August 2021. The cat samples submitted to this study were collected for the purpose of routine health monitoring by a veterinary hospital service at the Kasetsart University, Veterinary Teaching Hospital, Bangkok, Thailand.

### Sample size calculation

The sample size for this study was calculated using the below formula [19].

n = 1.962 P(1-P)/d^2^

The proportion (P) and maximum tolerated error (d) obtained from research article regarding the prevalence of factors associated with FeLV and FIV in cats from the Khon Kaen Province, Thailand [22]” The following sample sizes were calculated for FIV positive (FIV+), prevalence 6.1% (p = 0.061, d = 0.025, n = 353), for FeLV positive (FeLV+), prevalence 3.1% (p = 0.031, d = 0.02, n = 289), but not identified coinfection with both viruses. However, to increase the precision, a higher number of cats were sampled 480 cats based on the sample size calculated for FIV infection.

### Study design and sample collection

Overall, 480 cats were screened for feline retroviruses. The patient history was also obtained from each cat to identify any medical concern in their earlier life and at the time of sampling. A general physical examination was conducted. Whole blood samples (approximately 2–3 mL) were collected from each cat through venipuncture of the cephalic or jugular vein. Half of the blood samples were stored in tubes with ethylenediaminetetraacetic acid for hematologic analysis, and the other half were collected in serum-separating tubes for chemistry analysis. The tests were conducted as part of a routine animal examination.

### Feline retrovirus testing and risk factor assessment

The FIV and FeLV statuses were first tested using a lateral flow chromatographic immunoassay rapid kit (Witness FeLV-FIV [Zoetis]; United States). Samples with positive results were retested using a commercial rapid ELISA kit (SNAP FIV/FeLV Combo Test [IDEXX], Ludwigsburg, Germany). Both assays tested for the presence of the FeLV p27 antigen and FIV antibodies, which can help assess the current stage of infection. Age, gender, health, neuter status, body condition score, ownership, lifestyle, accommodation type, household type, behavior, and breed were recorded to evaluate risk factors associated with FIV and FeLV infections. A small proportion of the cats (19.16%, 92/480) was vaccinated and had received a complete course of vaccinations against FeLV or FIV; however, blood samples were collected before vaccinations.

### Laboratory examination

Hematologic and serum chemistry analyses were performed using an automated cell counter (CELL-DYN 3700; Abbott Laboratories; USA) and an automated chemical analyzer (Hitachi High-Technologies Co., Japan), respectively. A blood smear of each sample was evaluated after staining with Giemsa to visualize cell morphology and detect blood parasite infections. For all results, hematologic and biochemical parameters were compared with the laboratory reference values.

### Statistical analysis

All statistical analyses were performed using a standard software (STATA version 15.1; Stata Corporation, Texas, United States). Hematological and biochemical parameters were tested for normal distribution using the Shapiro-Wilk test. Data are expressed as median and range for non-normal distributed data. A comparison of all groups was performed using the Kruskal-Wallis test. Between-group analyses were conducted using the two-sample Wilcoxon rank-sum (Mann–Whitney) test. Risk factors were tested for the central limit theorem and odds ratio (OR) was determined through multinomial logistic regression analysis. Comparisons of the OR between the experimental and reference categories were considered significant at p < 0.05 and highly significant at p ≤ 0.001.

## Results

### Descriptive characteristics and risk factors

Of the 480 cats, 113 tested positive, with 8.3% FIV+, 12.5% FeLV+, and 2.7% positive for both viruses (FeLV/FIV+). Overall, 86 FIV/FeLV-negative cats with no clinical signs were considered the control group. The remaining 281 cats had other diseases and exhibited a wide range of clinical signs.

Overall, male cats were more likely to be infected with FIV, FeLV, and coinfected with FeLV-FIV (55%, 58.3%, and 76.9%, respectively) than female cats (45%, 41.7%, and 23.1%, respectively) (Table-1).

**Table 1:** Descriptive characteristics of the FeLV+, FIV+, FeLV/FIV+cats, and controls.

1Variable	FeLV+ (n = 60)	FIV+ (n = 40)	FeLV/FIV+ (n = 13)	Controls (n = 86)
Sex, no. (%)				
Male	35 (58.3%)	22 (55.0%)	10 (76.9%)	51 (59.3%)
Female	25 (41.7%)	18 (45.0%)	3 (23.1%)	35 (40.7%)
Age (year)				
Median (range)	3.75 (0.3–8.1)	7.04 (1–17.25)	5.08 (1.08–13)	3 (0.4–11)
Breed, no. (%)				
Domestic shorthair	58 (96.7%)	39 (97.5%)	13 (100.0%)	76 (89.4%)
Persian	2 (3.3%)	1 (2.5%)	0	7 (8.2%)
American wirehair	0	0	0	2 (2.4%)
Body weight (kg)				
Mean (SD)	3.77 (1.10)	3.89 (1.19)	4.58 (1.39)	4.90 (1.29)
Body condition score				
Median (range)	3 (1–5)	3 (2–5)	3 (1–4)	4 (1–5)
Sterilization, no. (%)				
Neutered	25 (41.7%)	10 (25.0%)	6 (46.2%)	4 (14.3%)
Intact	33 (58.3%)	30 (75.0%)	7 (53.9%)	24 (85.7%)
Health status, no. (%)				
Healthy	2 (3.8%)	6 (16.2%)	0	68 (81.0%)
Sick	51 (96.2%)	31 (83.8%)	13 (100.0%)	16 (19.1%)
Ownership, no. (%)				
Pet	25 (64.1%)	25 (62.5%)	9 (69.2%)	75 (96.2%)
Stray	14 (35.9%)	15 (37.5%)	4 (30.8%)	3 (3.9%)
Lifestyle, no. (%)				
Indoor	19 (50.0%)	12 (30.0%)	0	16 (59.3%)
Outdoor	19 (50.0%)	28 (70.0%)	13 (100.0%)	11 (40.7%)
Habitat, no. (%)				
Townhouse	7 (18.9%)	6 (16.2%)	3 (25.0%)	8 (30.8%)
House	20 (54.1%)	27 (73.0%)	9 (75.0%)	15 (57.7%)
Apartment	10 (27.0%)	4 (10.8%)	0	2 (7.7%)
Market	0	0	0	1 (3.9%)
Household, no. (%)				
One cat	11 (29.7%)	14 (35.9%)	3 (23.1%)	2 (6.9%)
More than 1 cat	26 (70.3%)	25 (64.1%)	10 (76.9%)	27 (93.1%)
Behavior, no. (%)				
Non-aggressive	30 (81.1%)	16 (40.0%)	5 (38.5%)	24 (92.3%)
Aggressive	7 (18.9%)	24 (60.0%)	8 (61.5%)	2 (7.7%)

FeLV+=Feline leukemia virus positive, FIV+=Feline immunodeficiency virus positive

The median ages for the FeLV+, FIV+, FeLV/FIV+, and control groups were 3.75, 7.04, 5.08, and 3 years, respectively. Factors that significantly increased the risk of FeLV infections were outdoor access, adulthood, and male gender. In contrast, significant risk factors for FIV infections were most common among middle-aged to old cats (median age, 7.04 years). Intact cats were more likely to be infected with FeLV or FIV than neutered cats, and domestic shorthair cats were more susceptible to FIV and FeLV infections than Persian and American wirehair breeds (Table-1).

### Multinomial regression analysis

**Figure-1:**
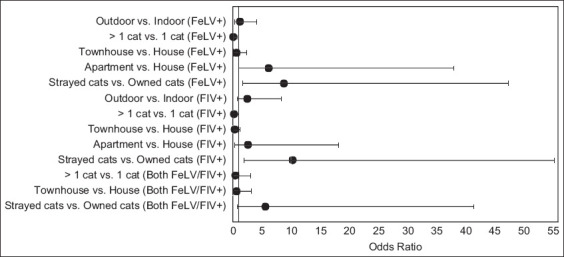
Odds ratios with 95% confidence interval of risk factors for FeLV+, FIV+, and FeLV/FIV+ cats. FeLV+=Feline leukemia virus positive and FIV+=Feline immunodeficiency virus positive.

Multinomial logistic regression was used to analyze the clinical data obtained from 113 positive cats (60 FeLV+, 40 FIV+, and 13 FeLV/FIV+) and 86 FeLV/FIV− controls. Data related to risk factors, including sterilization, health status, ownership, lifestyle, habitat, household, and behavior, are presented in Table-2. The estimated OR and 95% CIs of the factors associated with FeLV+, FIV+, and FeLV/FIV+ cats are depicted in Figure-1.

**Table 2:** Multivariate analysis comparing risk factors associated with FeLV+, FIV+, and FeLV/FIV+cats.

Variable	FeLV+				FIV+				FeLV/FIV+			
		
	n (%)	OR	95% CI	p-value	n (%)	OR	95% CI	p-value	n (%)	OR	95% CI	p-value
Lifestyle												
Indoor	19 (50.0)	1.0	(Reference category)	0.757	12 (30.0)	1.0	(Reference category)	0.132	0	1.0	(Reference category)	
Outdoor	19 (50.0)	1.22	0.35–4.27		28 (70.0)	2.56	0.75–8.70		13 (100.0)	N/A	N/A	
Household												
One cat	11 (29.7)	1.0	(Reference category)	0.041*	14 (35.9)	1.0	(Reference category)	0.05*	3 (23.1)	1.0	(Reference category)	0.457
Multi-cats	26 (70.3)	0.17	0.03–0.93		25 (64.1)	0.19	0.03–1.06		10 (76.9)	0.45	0.06–3.62	
Habitat												
House	20 (54.1)	1.0	(Reference category)	0.676	27 (73.0)	1.0	(Reference category)	0.152	9 (75.0)	1.0	(Reference category)	0.776
Townhouse	7 (18.9)	0.73	0.17–3.12	0.05*	6 (16.2)	0.33	0.07–1.51	0.339	3 (25.0)	0.76	0.12–4.83	
Apartment	10 (27.0)	6.43	1.00–41.31		4 (10.8)	2.61	0.36–18.71		0	N/A	N/A	
Market	0	N/A	N/A		0	N/A	N/A		0	N/A	N/A	
Owner ship												
Owned cats	25 (64.1)	1.0	(Reference category)	0.011*	25 (62.5)	1.0	(Reference category)	0.009*	9 (69.2)	1.0	(Reference category)	0.091
Strayed cats	14 (35.9)	9.17	1.67–50.48		15 (37.5)	9.42	1.75–50.66		4 (30.8)	5.81	0.75–44.81	
Sterilization												
Neuterized	33 (58.3)	1.0	(Reference category)	0.093	30 (75.0)	1.0	(Reference category)	0.598	7 (53.9)	1.0	(Reference category)	0.120
Intact	25 (41.7)	3.67	0.81–16.73		10 (25.0)	1.53	0.31–7.41		6 (46.2)	4.24	0.68–26.30	

* Significant difference, comparison of odds ratio between experimental categories and reference category. Odds ratios are calculated using multinomial logistic regression analysis. p < 0.05 was considered statistically significant. N/A is not applicable. FeLV+=Feline leukemia virus positive, FIV+=Feline immunodeficiency virus positive,

OR = Odds ratio, CI=Confidence interval

The OR of FeLV infection in cats living in a household with more than one cat was 0.17 (95% CI: 0.03–0.93, p < 0.05), lower than that for a household with only one cat. Similarly, the OR of FeLV infection in stray cats was 9.42 (95% CI: 1.75–50.66, p < 0.05) higher than that in pet cats. Cats living in apartments showed the highest OR of 6.43 compared with cats living in houses and townhouses. The OR of infection with FIV was significantly higher for multi-cat households at 0.19 (95% CI: 0.03–1.06, p < 0.05) than single-cat households. The OR of stray cats was 9.17, indicating that they were more likely to be infected with FIV (OR = 0.19, 95% CI: 0.03–1.06, p < 0.009) than owned cats. No statistically significant differences between intact and neutered cats were associated with FIV or FeLV infections (p = 0.093). Compared with cats kept indoors, those with known outdoor exposure had a higher OR for FIV infection than FeLV infection without significant differences (p = 0.757) (Table-2). No factor was significantly associated with FeLV-FIV coinfection. No statistically significant differences between intact and neutered cats were associated with FIV+ (p = 0.598) and FeLV+ (p = 0.093) (Table-2).

### Clinical signs

Clinical signs for both FeLV and FIV infections were variable (Table-3). Among the 60 FeLV+ cats, 21.7% (13/60) showed pale mucous membranes, 15% (9/60) had anemia, 15% (9/60) had mediastinal lymphoma, and 15% (9/60) had lymph node enlargement. The major clinical signs of 40 FIV+ cats included stomatitis (15%, 6/40), dyspnea (12.5%, 5/40), anemia (10%, 4/40), and lymph node enlargement (10%, 4/40). Of the 13 FIV/FeLV+ cats, 69.3% (9/13) presented with clinical alterations associated with virus infection, such as anemia (23.1%, 3/13), bacterial infections (28.57%, 4/13), and pale mucous membrane (15.4%, 2/13). Health status was evaluated based on the clinical records and physical examinations.

**Table 3:** The proportion of clinical signs associated with FeLV+, FIV+, and FeLV/FIV+cats.

Clinical signs, n (%)	FeLV+	FIV+	Both+	Clinical signs, n (%)	FeLV+	FIV+	Both+
					
	n = 60	n = 40	n = 13		n = 60	n = 40	n = 13
Ophthalmological sign				Immune system			
Conjunctivitis	3 (5.0)	-	1 (7.7)	Mediastinal lymphoma	9 (15.0)	-	-
Ocular discharge	1 (1.7)	1 (2.5)	1 (7.7)	T-cell lymphoma	3 (5.0)	2 (5.0)	1 (7.7)
Chemosis	-	-	1 (7.7)	B-cell lymphoma	-	1 (2.5)	-
Chorioretinitis	-	1 (2.5)	-	Lymph node enlargement	9 (15.0)	4 (10.0)	1 (7.7)
Urinary system				Integument system			
Urinary incontinence	-	2 (5.0)	-	Dermatitis	1 (1.7)	-	1 (7.7)
Nephritis	-	1 (2.5)	1 (7.7)	Otitis externa	1 (1.7)	-	-
Proteinuria	-	1 (2.5)	-	Abscess	-	2 (5.0)	-
Chronic kidney disease	-	1 (2.5)	-				
Respiratory system				Gastrointestinal system			
Nasal discharge	3 (5.0)	1 (2.5)	-	Stomatitis	6 (10.0)	6 (15.0)	-
Pleural effusion	8 (13.3)	4 (10.0)	-	Vomit	6 (10.0)	1 (2.5)	1 (7.7)
Sneezing	-	2 (5.0)	-	Regurgitation	1 (1.7)	-	-
Dyspnea	6 (10.0)	5 (12.5)	1 (7.7)	Gingivitis	3 (5.0)	3 (7.5)	1 (7.7)
Tracheitis	1 (1.7)	-	-	Peritonitis	1 (1.7)	-	-
Rhinitis	1 (1.7)	-	-	Enteritis	1 (1.7)	-	-
Pneumonia	1 (1.7)	2 (5.0)	-	Diarrhea	-	2 (5.0)	-
Lung tumor	2 (3.3)	-	-	Halitosis	1 (1.7)	-	-
Non-productive cough	1 (1.7)	2 (5.0)	1 (7.7)	Ascites	-	3 (7.5)	-
Hydrothorax	1 (1.7)	-	-	Constipation	-	1 (2.5)	-
Nasal cryptococcosis	-	1 (2.5)	-	Hepatic disease	1 (1.7)	1 (2.5)	-
Epistaxis	-	3 (7.5)	-				
Cardiology system				Endocrine system			
Hypertrophic	-	1 (2.5)	-	Polyuria/polydipsia	-	1 (2.5)	-
cardiomyopathy Cardiac arrhythmia	-	1 (2.5)	-	Diabetes mellitus	1 (1.7)	-	-
Neurology system				Bone/muscular system	-	1 (2.5)	-
Seizure	1 (1.7)	-	-	Hip dysplasia			
Hematology system				Non-specific sign			
Jaundice	1 (1.7)	-	1 (7.7)	Pyrexia	3 (5.0)	1 (2.5)	-
Pale mucous membrane	13 (21.7)	-	2 (15.4)	Depress	9 (15.0)	4 (10.0)	-
Non-regenerative anemia	1 (1.7)	-	-	Anorexia	14 (23.3)	4 (10.0)	2 (15.4)
Thrombocytopenia	1 (1.7)	1 (2.5)	1 (7.7)	Dehydrate	9 (15.0)	4 (10.0)	-
Neutropenia	-	2 (5.0)	-	Fever	-	1 (2.5)	-
Anemia	9 (15.0)	4 (10.0)	3 (23.1)	Weight loss	15 (25.0)	17 (42.5)	5 (38.5)
Eosinophilic granuloma	-	1 (2.5)	-	Lethargy	-	2 (5.0)	-

FeLV+=Feline leukemia virus positive, FIV+=Feline immunodeficiency virus positive

### Laboratory values

The median and range of different variables for complete blood count (CBC) and serum biochemistry are presented for all groups in Table-4. Most retrovirus-infected cats showed hematologic and biochemical results within the reference range. For CBC analysis, results for all blood parameters fell within the reference range for the control cats. Compared with uninfected cats, the results of red blood cell (RBC) tests were significantly different among FeLV+, FIV+, and FIV/FeLV+ cats for hemoglobin level; packed cell volume (PCV), RBC count; and mean corpuscular volume, mean corpuscular hemoglobin concentration, mean corpuscular hemoglobin, and red cell distribution width values (p ≤ 0.001). FeLV+ or FIV+ cats had significantly lower hemoglobin level; PCV, RBC counts; and RBC indices as well as lower monocyte, eosinophil, and platelet counts compared with control cats. Moreover, the risk of lymphopenia was higher in coinfected cats than in control cats. A comparison between FIV+ and FeLV+ cats revealed that FeLV+ cats were more likely to have low RBC and PCV counts but higher RBC indices than FIV-infected cats (p < 0.0001). In contrast, when compared with control cats, the white blood cell (WBC), neutrophil, and lymphocyte counts showed no significant difference between FeLV+ and FIV+ cats. However, the median values of neutrophil counts were higher in FIV+, FeLV+, and FeLV/FIV+ cats than in control cats. The monocyte, eosinophil, and platelet counts were significantly different in FeLV+, FIV+, and FeLV/FIV+ cats (p = 0.0205, 0.0042, and 0.0128, respectively) compared with control cats. The risk of thrombocytopenia was higher in cats with both infections than in control cats (p < 0.0128). No statistically significant differences were found in WBC count (p = 0.5453), neutrophil count (p = 0.0673), lymphocyte count (p = 0.2839), blood urea nitrogen (BUN) (p = 0.2033), or creatinine level (p = 0.0622) between any retrovirus group and controls; however, slightly higher values were observed in the control cats.

**Table 4:** Laboratory values and comparison of medians values between either FeLV+or FIV+, FeLV+, and FIV+ (i.e., both) and control cats.

Variables	Control	FeLV+	FIV+	Both FeLV/FIV+	p-value

	Median (range)				
Hemoglobin, g/dL	12.75 (8.5–15.9)^a,b,c^	9.65 (1.93–16.9)^a^	10.5 (5.37–14.4)^b^	9.25 (3.04–13)^c^	0.0001*
PCV, %	38.2 (26.2–50.0)^a,b,c^	30.85 (7.13–54.6)^a^	32.7 (17–41.2)^b^	30.3 (7.19–40.3)^c^	0.0001*
RBC, 10^6^/mL	8.15 (5.69–11.7)^a,b,c^	5.41 (0.99–10.2)^a,d^	6.84 (3.43–10.3) ^b,d,e^	5.49 (1.08– 7.89)^c,e^	0.0001*
MCV, fL	48.15 (31.8–65.0)^a,b^	52.88 (3.97–83.08) ^a,c,d^	49 (15.1–73.01)^c,e^	56.23 (48.42–79.45)^b,d,e^	0.0001*
MCHC, g/dL	32.96 (29.98–35.9) ^a,b,c^	31.84 (15.3–35.9)^a^	32.12 (29.32–37.5)^b^	31.92 (28.58–42.28)^c^	0.0002*
MCH, pg	15.6 (11.3–18.4)^a,b^	17.1 (8.99–33.4)^a,c,d^	15.81 (9.34–23.48)^c^	17.3 (15.34–28.15)^b,d^	0.0001*
RDW, %	20.1 (11.8–25.6)^a,b^	21.5 (16.5–60.6)^a,c^	19.8 (16–29.2)^c,d^	22 (18.7–51.5)^b,d^	0.0020*
WBC, 10^3^/mL	10.7 (4.72–33.0)	10.65 (1.98–37.6)	10.2 (3.57–41.2)	6.99 (1.38–36.0)	0.5453
Neutrophil, 10^3^/mL	72 (38–95)	81.5 (10–97)	77.5 (7–95)	78 (60–97)	0.0673
Lymphocyte, 10^3^/mL	18.5 (3–60)	13 (1–82)	12 (1–81)	16 (3–30)	0.2839
Monocyte, 10^3^/mL	4 (1–30)^a,b^	2 (0–10)^a,c^	5 (0–12)^c^	1 (0–7)^b^	0.0205*
Eosinophil, 10^3^/mL	5 (1–18)^a,b^	3 (0–17)^a^	3 (0–19)^b^	3 (0–9)	0.0042*
Platelet, 10^3^/mL	300 (53.5–1604)^a,b^	246 (7.2–712)^a^	300 (13.4–620)^c^	120 (28.4–620)^b,c^	0.0128*
BUN, mg/dL	24 (13–65.8)	24.5 (9–49)	28 (13–167)	21 (13–105)	0.2033
Creatinine, mg/dL	1.47 (0.73–56.0)	1.34 (0.69–2.22)	1.48 (0.68–9.47)	1.62 (0.74–3.33)	0.0622
ALT, IU/L	61 (17–716)	55 (18–415)	46 (18–461)	54 (25–132)	0.0776
Albumin, g/dL	3.5 (2.7–4.1)^a,b^	3.2 (2.2–4.2)^c^	3.4 (2.4–3.7)^a,c^	3.1 (2–7)^b^	0.0002*
γ-globulins, g/dL	4.3 (0–7.4)^a,b^	3.6 (2.6–6.2)^a,c,d^	5 (2.9–10.1)^b,c^	4.7 (0.3–5.4)^d^	0.0001*
Total protein	7.7 (6–9.6)^a^	7.1 (5.2–9.8)^a,b^	8 (5.9–12.7)^b^	7.4 (6.4–8.3)	0.0038*

a–e are upper alphabet that paired same letter for being significant difference of median between groups,

* Indicates a significant difference (the minimum significance value chosen was p < 0.05). A comparison of the median (range) of all groups was performed using the Kruskal–Wallis test, upper alphabet (paired same meaning significant difference) comparison of median between groups was performed using two-sample Wilcoxon rank-sum

(Mann–Whitney) test. p < 0.05 was considered statistically significant. PCV=Packed cell volume, RBC=Red blood cell, MC=Mean corpuscular volume, MCHC=Mean corpuscular hemoglobin concentration, MCH=Mean corpuscular hemoglobin, RDW=Red cell distribution width, WBC=White blood cell, BUN=Blood urea nitrogen, ALT=Alanine amino transferase, FeLV+=Feline leukemia virus positive, FIV+=Feline immunodeficiency virus positive

Serum chemistry analysis revealed that the albumin, globulin, and total protein levels were statistically significantly different in FeLV+, FIV+, and FeLV/FIV+ cats (p = 0.0002, 0.0001, and 0.0038, respectively) compared with control cats. Serum levels of total protein and g-globulin were significantly higher and serum albumin level was significantly lower in FIV+ cats compared with control cats. The levels of total proteins, albumin, and g-globulins were significantly lower in FeLV+ cats than in control cats. Cats with FeLV, FIV, and FeLV/FIV infections had significantly higher alanine aminotransferase (ALT) levels (p = 0.0776) than control cats.

## Discussion

In the present study, the infection rates for FIV, FeLV, and FeLV/FIV were 8.3%, 12.5%, and 2.7%, respectively, in Bangkok, Thailand. In a similar study conducted in Thailand, the prevalence rates of FIV infection, FeLV infection, and FeLV-FIV coinfection in 2015 and 2022 were 5.4%, 16.5%, and 3.5% [17] and 5.8%, 4.2%, and 0.4%, respectively [13]. Sprißler *et al*. [13] investigated 260 healthy cats with retrovirus infection in North, Northeast and Central Thailand. However, only a few studies have investigated the risk factors for FIV and FeLV infections in Thailand [17, 22]. In this study, the infection rate of FeLV was remarkably higher than that of FIV, which was consistent with the results of the previous studies [5, 17]. The variation between these studies may be due to the differences in the recruitment process, sample size, or factors such as geographic distribution, cat behavior, and cat health status [23, 24]. Although FeLV and FIV vaccines are available in Thailand, they are still not extensively used by Thai veterinarians due to the lack of updated information regarding the prevalence of the infections and the cost of vaccination. The high prevalence of both viral infections observed in this study might be because only a small proportion of the enrolled cats (19.16%, 92/480) were vaccinated and their vaccinations were not regular or up-to-date. Therefore, the vaccination data collected along with the sampling of the cats indicated that there is a need for improvement in vaccine administration among the cat population of Thailand.

Based on the combination of FeLV antigen/FIV antibody rapid test kits, FeLV or FIV infections were significantly higher in infected cats than in non-infected cats. These observations are similar to those of the previous studies conducted in Bangkok and its vicinity [17, 25]. Regarding risk factors in this study, multi-cat households and stray cats were more likely to be positive for FIV than FeLV, which may be because housing multiple cats causes stress among them as they fight for individual space. In addition, cats and kittens living in larger multi-cat households that lack proper hygiene can lead to the immunosuppressive effects of stress, resulting in the reactivation of some infections and increased susceptibility to new infections [26]. Male cats are more prone to infection than female cats for both viruses because males are more likely to live outdoor and sometimes fight with other males [14, 27]. Further, a high prevalence of retroviral infections is associated with domestic shorthair cats, the most common breed that originated in Thailand. The infection rates for both viruses differed according to age, such that FIV infection occurred more frequently in older cats and rarely in young cats, whereas FeLV was most prevalent in cats aged 6 years. These findings are consistent with those of other studies conducted in Thailand and other countries [28, 29]. According to the multivariate analysis, no other risk factor was significantly associated with a higher risk for FIV or FeLV infections.

Regarding laboratory parameters, all cats positive for retroviruses showed some hematologic abnormalities, such as anemia, thrombocytopenia, neutrophilia, and lymphopenia. This finding is consistent with the low blood cell counts reported in other studies [30–34], which may result from direct cytopathic effects on the bone marrow or caused by mechanisms involved in chronic inflammation [31, 34, 35]. In blood serum tests, total protein, albumin, and globulin levels were statistically significantly different in control cats compared with FeLV- and FIV-infected cats. These values were significantly lower in FeLV-infected cats than in the control cats. This finding is consistent with a report, which stated that FeLV-infected cats are more likely to have low total protein, albumin, and globulin levels than non-infected cats [31]. Other studies reported that the BUN, creatinine, and serum ALT levels were significantly lower in FeLV-infected cats than in non-infected cats [36, 37]. Although the present study showed no statistically significant association between infected and control cats concerning these three values, the BUN and creatinine levels were higher in cats infected with FeLV than in those infected with FIV. Cats infected with FeLV may develop opportunistic infections that result in bacterial colonization of the upper and lower urinary tract [38, 39]. Even though several cats that test positive for FeLV and/or FIV do not present with abnormalities on physical examination, CBC, chemistry, and urine tests should be performed to check for underlying abnormalities that could signal the presence of FeLV- or FIV-related disorders.

This study is based on a combined analysis of retrospective and present data. Because of the extensive number of cases, missing data did not impact the significance of the presented results. A limitation of the study was that FIV/FeLV+ test results were not confirmed using Western blot for FIV infection or immunofluorescent antibody test, virus isolation, or PCR for FeLV infection. These confirmatory tests should be performed in future studies. Another limitation was that some owners declined the recommended care because of limited financial ability and the opinion that such care is nonessential. Thus, improving communication between veterinarians and cat owners may help increase compliance regarding vaccinations.

## Conclusion

Of the 480 cats in the study sample, 40 (8.3%) tested positive for FIV antibodies, 60 (12.5%) for FeLV antigen, and 13 (2.7%) for FIV-FeLV coinfection. The major risk factors for FeLV and FIV infections included sex, age, behavior, sickness, outdoor lifestyle, and multi-cat households. According to the present study, the combined use of confirmatory testing and vaccination is an important tool to decrease the infection rate of feline retrovirus in Bangkok, Thailand. This study confirmed that laboratory results help veterinarians monitor disease development and aid in the diagnosis of cats infected with retroviruses. Further research should be targeted toward determining the correlation between clinical signs in and laboratory data of FIV- and FeLV-infected cats.

## Authors’ Contributions

JR: Conceived and supervised the study and manuscript editing. OR: Designed and conducted the study, interpreted the results, and drafted the manuscript. TJ: Performed statistical analysis. SH, CB, and KC: Study design and interpretation of the data. All authors have read and approved the final manuscript.
